# Exosomal MiR-199a-5p Inhibits Tumorigenesis and Angiogenesis by Targeting VEGFA in Osteosarcoma

**DOI:** 10.3389/fonc.2022.884559

**Published:** 2022-05-16

**Authors:** Lu Zhang, Hongxin Cao, Guanghui Gu, Dehui Hou, Yunhao You, Xiang Li, Yunzhen Chen, Guangjun Jiao

**Affiliations:** ^1^ Department of Orthopedics, Qilu Hospital of Shandong University and Spine and Spinal Cord Disease Research Center, Shandong University, Jinan, China; ^2^ Department of Medical Oncology, Qilu Hospital of Shandong University, Jinan, China; ^3^ Key Laboratory of Chemical Biology (Ministry of Education), Institute of Biochemical and Biotechnological Drug, School of Pharmaceutical Sciences, Cheeloo College of Medicine, Shandong University, Jinan, China

**Keywords:** osteosarcoma, exosome, angiogenesis, miR-199a-5p, VEGFA

## Abstract

**Background:**

Osteosarcoma (OS) is the most common primary bone malignancy in children and adolescents. microRNAs have been found to play a vital role in tumor angiogenesis. Here, we investigated the effects of miR-199a-5p on tumor growth and angiogenesis in osteosarcoma. Furthermore, the underlying molecular mechanisms and signaling pathways were explored.

**Methods:**

The datasets were extracted from the Gene Expression Omnibus and the differentially expressed miRNAs (DEmiRNAs) were screened out by the GEO2R online platform. The potential target genes were predicted using the miRTarBase database. The predicted target genes were further analyzed by Gene Ontology and pathway enrichment analysis and a regulatory network of DEmiRNAs and their target genes was constructed. In addition, the effects of osteosarcoma cell derived exosomal miR-199a-5p on the proliferation, migration and neovascularization of HUVECs were evaluated by conducting EdU assays, Transwell experiments and tube formation assays. A dual-luciferase reporter assay was performed to detect whether VEGFA was the direct target of miR-199a-5p. Furthermore, *in vivo* xenograft models were established to further investigate the intrinsic role of miR-199a-5p in osteosarcoma tumorigenesis and angiogenesis.

**Results:**

A total of 149 DE-miRNAs were screened out, including 136 upregulated miRNAs and 13 downregulated miRNAs in human osteosarcoma plasma samples compared with normal plasma samples. A total of 1313 target genes of the top three upregulated and downregulated miRNAs were predicted. In the PPI network, the top 10 hub nodes with higher degrees were identified as hub genes, such as TP53 and VEGFA. By constructing the miRNA-hub gene network, we found that most of hub genes could be potentially modulated by miR-663a, miR-199a-5p and miR-223-3p. In addition, we found that the expression level of miR-199a-5p in exosomes derived from osteosarcoma cells was remarkably higher than the osteosarcoma cells, and the exosomes derived from osteosarcoma cells were transported to HUVECs. Overexpression of miR-199a-5p could significantly inhibited HUVEC proliferation, migration and neovascularization, whereas downregulation of miR-199a-5p expression exerted the opposite effect. Moreover, the *in vivo* results verified that overexpression of miR-199a-5p in osteosarcoma cells could suppress the growth and angiogenesis of tumors.

**Conclusion:**

Our results demonstrated that miR-199a-5p could be transported from osteosarcoma cells to HUVECs through exosomes, subsequently targeting VEGFA and inhibiting the growth and angiogenesis of osteosarcoma. Therefore, miR-199a-5p may act as a biomarker in the diagnosis and treatment of osteosarcoma.

## Introduction

Osteosarcoma is the most common primary malignant bone tumor in children and adolescents, and its incidence reaches a second peak in adults over 65 years old (1). Although modern multimodality therapy has meaningfully improved tumor resectability and long-term prognosis in these patients, 25-35% of patients with primary nonmetastatic disease subsequently develop metastasis, which is still the leading cause of death (2). In the process of tumor metastasis, metastatic colonization is the speed limiting point of the cascade reaction of tumor metastasis. Studies have shown that if disseminated tumor cells cannot stimulate new angiogenesis, the process of metastatic colonization will be inhibited, and *vice versa (* 3). Therefore, elucidating the molecular mechanism of angiogenesis in the process of osteosarcoma metastasis and finding potential therapeutic targets will contribute to the improvement of effective therapies for osteosarcoma.

The tumor microenvironment plays a crucial role in the occurrence and progression of tumors. Many factors are involved in the regulation of the tumor microenvironment. Recently, more and more attention has been given to the role of exosomes in the tumor microenvironment. Exosomes derived from tumor cells can regulate the tumor environment in a variety of ways to promote tumor progression. It has been proven that the exosome volume secreted by tumor cells is much higher than that secreted by nontumor cells (4). Many studies have shown that miRNAs in exosomes are involved in multiple stages of tumor occurrence and development, including chemotherapy resistance, immunosuppression and the formation of tumor metastasis (5, 6). Therefore, exploring the role of miRNAs from exosomes in the development of osteosarcoma will contribute to finding new diagnostic markers or therapeutic targets.

MicroRNAs (miRNAs) are small noncoding RNAs that can regulate gene expression by direct cleavage of targeted messenger RNAs (mRNAs) or by inhibiting translation through binding to targeted mRNAs at the 3′ untranslated regions (UTRs) (7). MiRNAs are significant contributors to the initiation and progression of tumor growth because of their ability to inhibit the expression of oncogenes and tumor suppressor genes. With the further study of microRNAs, microRNAs have been found to play an important role in tumor angiogenesis. MiR-126, miR-378, miR-296, and miR-210 have been proven to promote angiogenesis *in vivo* and *in vitro (* 8). It is widely believed that miRNAs affect the function of endothelial cells by regulating the expression of angiogenic factors such as VEGF in tumor cells (9). Emerging evidence shows that tumor cells secrete extracellular vesicles to transfer miRNA to vascular endothelial cells, regulate their gene transcription, and affect the function of vascular endothelial cells, thereby affecting tumor angiogenesis and metastasis (10, 11).

Studies have shown that inhibiting the expression of miR-199a-5p can promote the invasion of human hepatocellular carcinoma (12). MiR-199a-5p can inhibit the proliferation and invasion of ovarian cancer cells through the NF-κB1 pathway (13). MiR-199a-5p suppresses glioma progression by inhibiting MAGT1 (14). The expression level of miR-199a-5p in patients with endometriosis is significantly lower than that in normal people, and further studies have found that miR-199a-5p promotes the development of endometriosis by regulating VEGFA in endometrial mesenchymal stem cells (15). However, the biological mechanism of miR-199a-5p in angiogenesis of osteosarcoma is not well understood. The investigation of the effects of miR-199a-5p and its target protein on the angiogenesis of osteosarcoma may lead to new perspectives for clinical trials of gene therapy.

In this study, we screened the differentially expressed miRNAs in osteosarcoma using the miRNA expression profile of GSE65071. Differential expression of miRNA target genes and their potential functions were predicted by functional enrichment and pathway enrichment analysis. Then, we constructed regulatory networks of osteosarcoma-related miRNAs and their target genes. We further verified the mechanism of miR-199a-5p and its target genes in the angiogenesis of osteosarcoma. HUVEC proliferation, migration, invasion and new vessel formation are significantly inhibited by miR-199a-5p both *in vitro* and *in vivo*. In addition, miR-199a-5p inhibits transduction of the HIF-1α/VEGF signaling pathway. Taken together, our findings provide valuable clues to the pathogenesis of osteosarcoma and provide a promising target for the development of effective treatment in the future.

## Materials and Methods

### MiRNA Microarray Analysis

The miRNA dataset GSE65071 (16) was downloaded from the GEO (Gene Expression Omnibus) database (https://www.ncbi.nlm. nih.gov/geo/). GEO2R (http://www.ncbi.nlm.nih.gov/geo/geo2r), a web-based application, contributes to filtrating differentially expressed miRNAs (DEmiRs) through Bioconductor’s GEO Query and limma R software (11). Therefore, in the present study, the DEmiRs between the osteosarcoma samples and control samples were screened out by the GEO2R online platform with cutoff criteria of P<0.05 and |log2 fold‐change| ≥1‐fold. The genes targeted by DEmiRs were predicted using the miRTarBase database (17) (http://mirtarbase.cuhk.edu.cn/). Enrichment analysis of biological processes, molecular functions and cellular components of target genes was carried out online through the Enrichr website (http://amp.pharm.mssm.edu/Enrichr/).

Online analysis was performed for the interaction of downstream target proteins through the STRING website (https://string-db.org/cgi/input.pl?sessionId=mQ0AB12ssIaK&input_page_show_search=on). The interaction network diagram of “miRNA-mRNA” was constructed by Cytoscape software (Version 3.7.1). The differences in the expression of target genes in osteosarcoma and normal tissue were calculated by using the R2 database (https://hgserver1.amc.nl/cgi-bin/r2/main.cgi) (Mixed Osteosarcoma - Aqeilan - 18 - MAS5.0 - u133p2). The prognostic analysis of target genes in osteosarcoma were calculated *via* R2 database (https://hgserver1.amc.nl/cgi-bin/r2/main.cgi) (Mixed Osteosarcoma (Mesenchymal) - Kuijjer - 127 - vst - ilmnhwg6v2). Set the threshold to Cox P value <0.05. The correlation coefficient was calculated using Spearman’s method.

### Cell Culture

The human osteosarcoma HOS and MG63 cell lines were purchased from the Shanghai Zhong Qiao Xin Zhou Biotechnology Company Limited. The cells were cultured with RPMI 1640 containing 10% FBS. Human umbilical vein endothelial cells (HUVECs) were purchased from Zhong Qiao Xin Zhou Biotechnology Co., Ltd. (Shanghai, China). HUVECs were cultured in endothelial cell medium containing 10% FBS, endothelial cell growth supplement and 1% double antibiotics (18). Both cell types were maintained in an incubator at 37°C and 5% CO_2_. HOS cells in the logarithmic growth phase and HUVECs at passages three through six were used in the studies.

### Isolation and Purification of Exosomes

Exosomes were isolated from the supernatant of osteosarcoma cells according to the previously described protocol (18). HOS cells at 80% confluence were cultured in complete medium for 48 hours, and then the medium was transferred to new test tubes, and centrifuged at 300 g for 10 minutes at 4°C. The supernatant was then centrifuged at 16,500 g at 4°C for 30 minutes to remove cell debris. The cell supernatant was filtered using a 0.22-μm filter to remove whole cells and excess cell debris. After that, the supernatant was transferred to new tubes and ultracentrifuged at 100,000 g at 4°C for 70 minutes to precipitate exosomes. Exosomes were identified by nanoparticle tracking analysis (NTA), transmission electron microscopy (TEM) and western blotting.

### Uptake of Osteosarcoma Exosomes by HUVECs

According to the manufacturer’s instructions, the osteosarcoma cell-derived exosomes were labeled with PKH26 (Sigma-Aldrich, Italy). In short, the exosomes were incubated with PKH26 for 10 minutes at room temperature. The labeled exosomes were washed twice in PBS, centrifuged, resuspended in low serum medium, and incubated with HUVECs at 4°C for 4 hours. HUVECs were stained with phalloidin (Solarbio Life Sciences, China), which binds F-actin with high affinity. Cell nuclei were stained with DAPI (Solarbio Life Sciences, China) and analyzed by confocal microscopy.

### EdU Assay

To examine the proliferation ability of HUVECs, the cells were seeded in 24 well medium containing 10% FBS for 24 hours. Then, the cells were cultured in serum-free medium, and mimics or inhibitors were added to the experimental group. After that, EdU labeling reagent (Ribio, Guangzhou, China) was added at a dilution of 1:1000. After 6 hours, the cells were fixed with paraformaldehyde for 30 minutes, and subsequently immersed in 2 mg/ml glycine solution for 5 minutes, and then incubated with 0.5% TritonX-100 in PBS at room temperature for 20 minutes. According to the instructions, a Cell-Light EdU Apollo567 *In Vitro* Kit (Ribbio, Guangzhou, China) was used to detect cells. The image was analyzed by ImageJ software.

### HUVEC Tube-Formation Assay

After dissolving the basement membrane matrix (Corning, Life Sciences, USA) at 4°C, it was infused into a 48-well plate at 100 μL per well and incubated at 37°C for 1 hour. Then, 200 μL of endothelial cell culture medium without FBS containing 2×10^4^ HUVECs were added to the plate. The control group was treated with medium only, and the test group was mixed with 100 μL of exosomes. The total length of tube branches and tubes was calculated using ImageJ software.

### Western Blot Analysis

Western blotting was performed as previously described (18). Briefly, a cell lysis buffer (RIPA buffer, Biomed, Shanghai, China) was used to obtain total protein from cultured cells. The proteins were separated by gel electrophoresis and transferred to a polyvinylidene fluoride (PVDF) membrane. After blocking it in 5% milk in phosphate-buffered saline with Tween 20 (w/v), the membrane was incubated with the primary antibody and the secondary antibody, respectively. The main antibodies used were monoclonal VEGFA antibody (ab46154 Abcam, Abcam, MA, USA) and β-actin antibody (AF7018, Affinity Biosciences, OH, USA). Then, the membranes were scanned, and protein levels were standardized to β-actin (1:1000) as an internal reference. The ChemiDoc Touch Gel Imaging System and Image Lab Touch software (Bio-Rad, CA, USA) were utilized to record and quantify the signal intensity.

### Invasion Assay

According to the manufacturer’s instructions, 8-μm pore filters were used for Transwell invasion assays (Corning, Bedford, USA) to determine the invasion. In brief, HUVECs (3×10^4^ cells) treated with miR-199a-5p mimics or inhibitor for 24 hours were washed and resuspended in RPMI 1640 medium supplemented with 0.1% BSA. The cells were then placed on the upper part of a 24-well Transwell insert coated with Matrigel^®^ (Corning, Bedford, US), while the lower chamber contained endothelial cell medium (ScienCell, USA). After 6 hours, the filter was removed, fixed and stained with crystal violet staining solution (0.1%, Solarbio Life Sciences, China).

### Tumor Xenograft Assay

Six-week-old BALB/c female athymic nude mice (Vitalriver, Beijing, China) were subcutaneously injected in the right flank using HOS cells with stable expression of lentivirus miR-199a-5p with stable expression of miR-199a-5p or HOS cells with lenti-miR-199a-5p-NC or HOS cells with PBS. Mice were humanely sacrificed on Day 28. Tumor samples were weighed and then processed for routine immunohistochemistry and western blot (19).

### Statistical Analysis

Graphpad prism 8 (Graphpad Software, San Diego, CA) was applied for statistical analysis in this study. All experiments were repeated at least three times. All values are expressed as the mean ± standard deviation. Analysis of variance was used for comparisons between multiple groups. Student’s t-test was used to compare two independent groups. For the data conforming to the normal distribution, we use the two way t-test for statistical analysis. A P value of <0.05 was considered statistically significant.

## Results

### Identification of DE−MiRNAs and Their Target Genes

**Figure 1:**
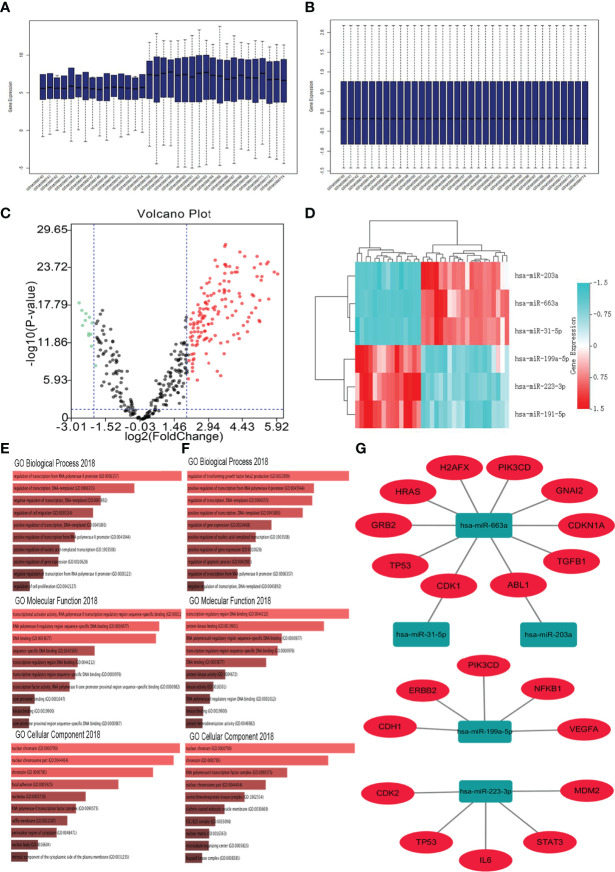
Identification of DE−miRNAs and their target genes. **(A)** GSE65071 data before normalization; **(B)** GSE65071 data after normalization; **(C)** Volcano plot of the DE-miRNAs. The black dots represent miRNAs that are not differentially expressed between human osteosarcoma plasma samples and normal plasma samples, and the red dots and green dots represent the upregulated and downregulated miRNAs in human osteosarcoma samples, respectively. **(D)** Heat map of the top three differentially expressed miRNAs. Red represents upregulated miRNAs. Cyan represents downregulated miRNAs. **(E)** GO functions for the target genes of the top three upregulated miRNAs; **(F)** GO functions for the target genes of the top three downregulated miRNAs. **(G)** The regulatory network between dysregulated miRNAs and hub genes.

The GSE65071 dataset (20 osteosarcoma plasma samples and 15 normal plasma samples) was first downloaded and preprocessed (normalized) (**Figures 1A, B**). After preprocessing and removing batch effects, we analyzed the DEmiRNAs of GSE65071 using the limma package using FDR (false discovery rate) P < 0.05 and |log2fold change (FC)| > 2 for upregulated genes and |log2fold change (FC)| < − 2 for downregulated genes as the cutoff criteria. Based on this analysis, a total of 149 miRNAs were found to be significantly differentially expressed in osteosarcoma samples more than twofold when compared to normal samples, including 136 upregulated and 13 downregulated miRNAs. For better visualization, the top three most upregulated miRNAs and top three most downregulated miRNAs are presented in **Table 1**. A total of 864 potential target genes were predicted for the three upregulated miRNAs (hsa-miR-663a, hsa-miR-31-5p, and hsa-miR-203a) and 449 genes were predicted for the three downregulated miRNAs (hsa-miR-199a-5p, hsa-miR-223-3p, and hsa-miR-191-5p) by using miRTarBase. A volcano plot of these DE-miRNAs is provided in **Figure 1C**. Cluster analysis of the top three DEmiRNAs (up- and downregulation) also revealed significant differences between osteosarcoma samples and normal samples (**Figure 1D**).

**Table 1:** The top three most miRNAs and top three most downregulated miRNAs between osteosarcoma and normal plasma samples.

miRNA_ID	adj.P.Val	P.Value	logFC	SEQUENCE
Upregulated miRNAs				
hsa-miR-663a	1.89E-22	1.20E-23	5.91579	aggcggggcgccgcgggaccgc
hsa-miR-31-5p	1.20E-22	5.97E-24	5.74147	aggcaagatgctggcatagct
hsa-miR-203a	8.64E-19	1.12E-19	5.51882	gtgaaatgtttaggaccactag
Downregulated miRNAs				
hsa-miR-199a-5p	2.40E-13	8.83E-14	-3.01081	cccagtgttcagactacctgttc
hsa-miR-223-3p	2.91E-18	4.43E-19	-2.72998	tgtcagtttgtcaaatacccca
hsa-miR-191-5p	4.05E-17	7.71E-18	-2.60448	caacggaatcccaaaagcagctg

### Enrichment Analysis of the Target Genes

Tree categories of GO functional annotation analysis were performed on these potential target genes, including biological process (BP), molecular function (MF) and cellular component (CC). As shown in **Figure 1E**, the enriched GO functions for target genes of the three upregulated miRNAs included the regulation of transcription from RNA polymerase II promoter, regulation of transcription, DNA templated, negative regulation of transcription, DNA templated, regulation of cell migration, and positive regulation of transcription, DNA templated in the BP category. For the MF items, these mRNAs were enriched in transcriptional activator activity, RNA polymerase II transcription regulatory region sequence-specific binding, RNA polymerase II transcription regulatory region sequence-specific DNA binding, and DNA binding (**Figure 1E**). Regarding CC, the DEmiRNAs were significantly enriched in nuclear chromatin, nuclear chromosome part, chromatin, focal adhesion, and nucleolus (**Figure 1E**). As shown in **Figure 1F**, the enriched GO functions for target genes of the three downregulated miRNAs included the regulation of transforming growth factor beta2 production, positive regulation of transcription from RNA polymerase II promoter, regulation of transcription, DNA templated, positive regulation of transcription, DNA templated, and regulation of gene expression in the BP category. For the MF items, these mRNAs were enriched in transcription regulatory region DNA binding, protein kinase binding, RNA polymerase II transcription regulatory region sequence-specific binding, and RNA polymerase II transcription regulatory region sequence-specific DNA binding (**Figure 1F**). Regarding CC, the DEmiRNAs were significantly enriched in nuclear chromatin, chromatin, RNA polymerase II transcription factor complex, and nuclear chromosome part (**Figure 1F**).

### Construction and Analysis of the PPI Network and miRNA−Hub Gene Network

To explore the interactions of the target genes, a PPI network based on the STRING database was constructed. The results showed that many of these target genes could interact with each other. The top ten hub genes listed in **Table 2** were screened out according to the node degree. For the upregulated miRNAs, the hub genes were TP53, PIK3CD, HRAS, CDKN1A, TGFB1, H2AFX, GRB2, ABL1, CDK1, and GNAI2. For the downregulated miRNAs, the hub genes were TP53, VEGFA, IL6, PIK3CD, STAT3, CDK2, NFKB1, CDH1, ERBB2, and MDM2. Among these genes, TP53 node degree is the highest. The results suggest that TP53 may be an important target correlated with osteosarcoma.

**Table 2:** Hub genes identified in the PPI interaction.

Upregulated miRNAs		Downregulated miRNAs	
Gene symbol	Degree	Gene symbol	Degree
TP53	26	TP53	96
PIK3CD	15	VEGFA	69
HRAS	14	IL6	61
CDKN1A	11	PIK3CD	55
TGFB1	10	STAT3	53
H2AFX	10	CDK2	52
GRB2	10	NFKB1	52
ABL1	9	CDH1	47
CDK1	9	ERBB2	43
GNAI2	8	MDM2	42

PPI, protein-protein interaction.

Subsequently, Cytoscape software was used to construct the miRNA-hub gene network, as shown in **Figure 1G**. From **Figure 1G**, we found that ten hub genes (TP53, PIK3CD, HRAS, CDKN1A, TGFB1, H2AFX, GRB2, ABL1, CDK1, and GNAI2) could be potentially modulated by upregulated miR-663a. One hub gene each may be regulated by miR-31-5p and miR-203a. Additionally, miR-199a-5p could potentially target five (VEGFA, PIK3CD, NFKB1, CDH1, and ERBB2) of ten hub genes. Another five hub genes (TP53, IL6, STAT3, CDK2, and MDM2) may be regulated by miR-223-3p. These data indicated that miR-663a, miR-199a-5p, and miR-223-3p might be three potential regulators in the development of osteosarcoma.

**Figure 2:**
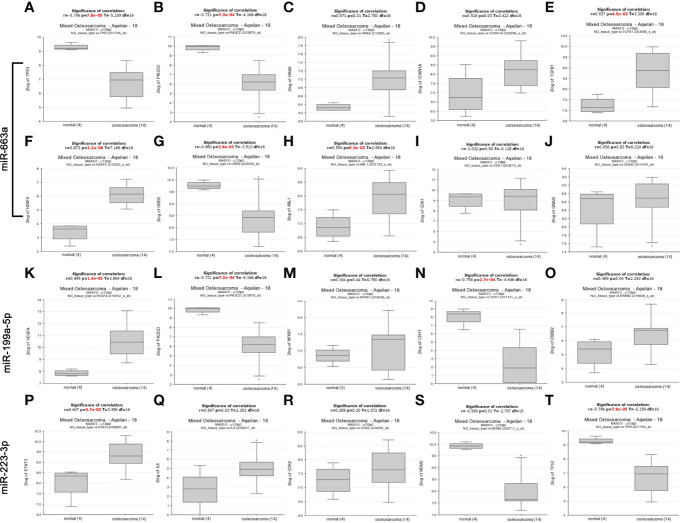
The mRNA expression of predicted targets of miR-663a, miR-199a-5p and miR-223-3p from the R2 database (https://hgserver1.amc.nl/cgi-bin/r2/main.cgi). **(A)** TP53 expression; **(B)** PIK3CD expression; **(C)** HRAS expression; **(D)** CDKN1A expression; **(E)** TGFB1 expression; **(F)** H2AFX expression; **(G)** GRB2 expression; **(H)** ABL1 expression; **(I)** CDK1 expression; **(J)** GNAI2 expression; **(K)** VEGFA expression; **(L)** PIK3CD expression; **(M)** NFKB1 expression; **(N)** CDH1 expression; **(O)** ERBB2 expression; **(P)** STAT3 expression; **(Q)** IL6 expression; **(R)** CDK2 expression; **(S)** MDM2 expression; **(T)** TP53 expression. The expression of HRAS, CDKN1A, TGFB1, H2AFX, ABL1, VEGFA, ERBB2, STAT3 and IL6 was significantly upregulated in osteosarcoma tissue compared with normal tissue whereas TP53, PIK3CD, GRB2, CDH1 and MDM2 were markedly downregulated.

In addition, we analyzed the difference of these 18 mRNA expression between osteosarcoma patients and normal human tissues through the database (https://hgserver1.amc.nl/cgi-bin/r2/main.cgi) (20). As showed in the **Figure 2**, the analytic results revealed that the expression of four (HRAS, CDKN1A, TGFB1, H2AFX and ABL1) of eight targets of miR-663a was significantly upregulated in osteosarcoma tissue compared with normal tissue whereas TP53, PIK3CD and GRB2 were markedly downregulated. It has been widely recognized that there is a negative correlation between miRNA expression and target gene expression. Therefore, among these detected genes, the significantly downregulated TP53, PIK3CD and GRB2 may be the most potential targets for the upregulated miR-663a. Likewise, these meaningfully upregulated genes including VEGFA and ERBB2 may be the most potential targets for the downregulated miR-199a-5p in theory. And STAT3 and IL6 may be the most potential targets for the downregulated miR-223-3p.

Next, we further evaluated the patients’ survival prognostic information of these 18 hub mRNAs in osteosarcoma using the R2 database (https://hgserver1.amc.nl/cgi-bin/r2/main.cgi) (20) as shown in **Figures S1**, **S2**. The results showed that the low expression of PIK3CD was associated with poor overall survival and metastasis free survival in patients with osteosarcoma. The high level of VEGFA expression was related with poor overall survival and metastasis free survival in osteosarcoma patients. In conclusion, among these detected genes, PIK3CD may be the most potential targets for the upregulated miR-663a. Similarly, VEGFA may be the most potential targets for the downregulated miR-199a-5p in theory.

### MiR-199a-5p Could be Transported Into HUVECs *via* Exosomes From Osteosarcoma Cells

**Figure 3:**
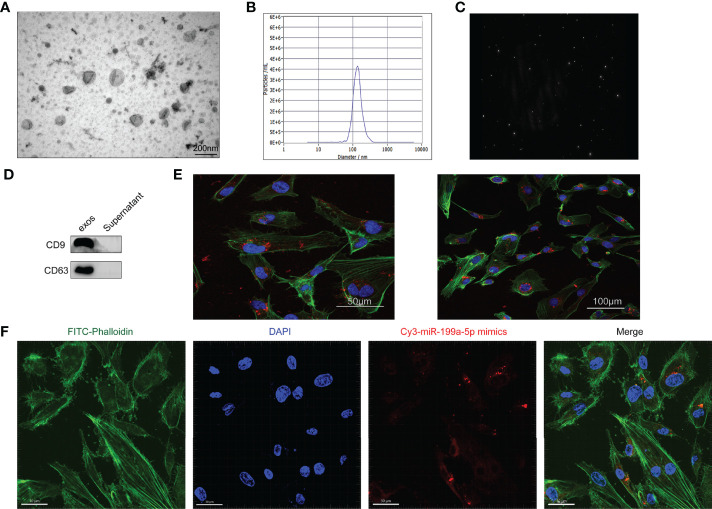
Characterization of Exosomes. **(A)** The morphology of HOS-Exosomes shown by TEM. **(B)** The particle size distribution in purified Hos-exosomes determined by NTA (nanoparticle tracking analysis). **(C)**, Image of the purified exosomes. **(D)** The surface markers (CD9 and CD63) of exosomes were detected by western blotting. **(E)** Laser scanning confocal microscopy analysis of PKH26‐labeled Hos-exosomes internalization by HUVECs. The red‐labeled exosomes were visible in the perinuclear region of recipient cells. **(F)** Laser scanning confocal microscopy analysis of HOS-exosomal miR-199a-5p-mimics-cy3 internalization by HUVECs.

To evaluate the effect of osteosarcoma exosomes on tumor angiogenesis, we first needed to determine whether the tumor exosomes were taken up by HUVECs. The extracted exosomes of HOS cells were characterized using TEM, NanoSight and western blotting. TEM images showed that the majority of the particles exhibited a cup- or round-shaped morphology (**Figure 3A**). As measured by NTA (nanoparticle tracking analysis), the diameter of the exosomes was approximately 122 nm (**Figures 3B, C**). The expression of the CD9 and CD63 proteins was detected using western blotting (**Figure 3D**). These results suggested that the extracted exosomes had characteristics that met widely accepted standards.

Additionally, to illuminate whether tumor-secreted exosomes were delivered to endothelial cells, exosomes derived from HOS cells were labeled with PKH67, and then the conditioned medium was collected and processed for exosomal purification. We then observed the transport of exosomes from osteosarcoma cells to HUVECs. After incubation with PKH67-labeled exosomes derived from HOS cells, PKH67 signals were localized in the cytoplasm of HUVECs (**Figure 3E**).

VEGFA is an important factor in the regulation of angiogenesis, and VEGFA is a potential target gene of miR-199a-5p, so we also detected whether miR-199a-5p was transported into HUVECs. As shown in **Figure 3F**, we found that HOS-exosomal miR-199a-5p-mimics-cy3 were internalized by HUVECs. Collectively, these data clearly demonstrated that miR-199a-5p secreted in the exosomes of osteosarcoma cells can be effectively transmitted into HUVECs.

### Exosomal MiR-199a-5p Derived From Osteosarcoma Cells Targets VEGFA in HUVECs

**Figure 4:**
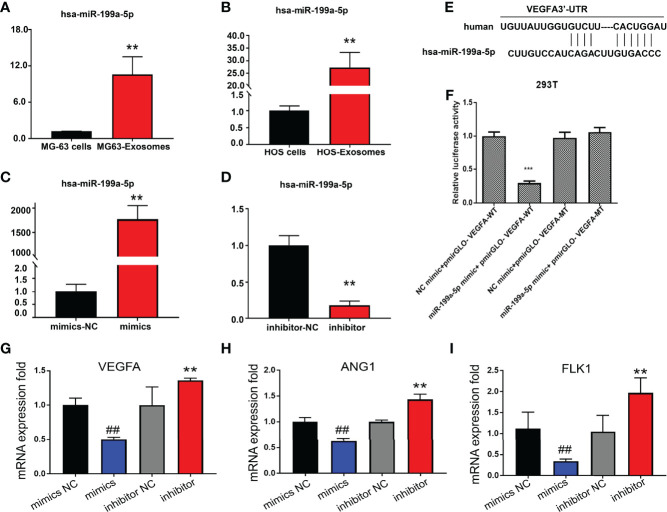
Detection of miR-199a-5p expression level. **(A)** Detection of miR-199a-5p expression in MG63 cells and exosomes. **(B)** Detection of miR-199a-5p expression in HOS cells and exosomes. **(C)** Detection of miR-199a-5p expression after mimic transfection of HUVECs. **(D)** Detection of miR-199a-5p expression after inhibitor transfection of HUVECs. **(E)** Prediction of the binding sites of hsa-miR-199a-5p and VEGFA mRNA. **(F)** Results of double luciferase reporter gene assay. **(G)** Detection of VEGFA expression after mimic or inhibitor transfection of HUVECs. **(H)** Detection of ANG1 expression after mimic or inhibitor transfection of HUVECs. **(I)** Detection of FLK1 expression after mimic or inhibitor transfection of HUVECs. **P < 0.01, ^##^P < 0.01, ***P < 0.001.

RT-PCR was applied to detect the expression of miR-199a-5p in osteosarcoma cells and their exosomes. The data indicated that the expression level of miR-199a-5p in exosomes derived from HOS and MG63 cells was remarkably higher than that in HOS and MG63 cells (**Figures 4A, B**). Additionally, miR-199a-5p mimics and inhibitor were transfected into HUVECs respectably.

It has been proven that miR-199a-5p can be transferred to HUVECs through exosomes, so we tested the effect of miR-199a-5p on HUVECs. We found that the expression of miR-199a-5p in HUVECs was significantly increased or decreased after transfection with mimics or inhibitor (**Figures 4C, D**).

According to our previous bioinformatics analysis, VEGFA was a candidate target of miR-199a-5p, whose mRNA 3’-UTR contains the miR-199a-5p interactive sequence (**Figure 4E**). To further clarify that VEGFA is a direct target of miR-199a-5p, we amplified the corresponding wild-type (WT) and mutant (MuT) 3’-UTRs and cloned them into the PGL3 luciferase reporter vectors. The relative luciferase activity of wild-type (WT) vectors was significantly diminished upon cotransfection with the miR-199a-5p mimic. However, the luciferase activity of the vectors harboring mutant (MuT) 3’-UTRs of these genes remained unchanged (**Figure 4F**). In addition, overexpression of miR-199a-5p using a miR-199a-5p mimic significantly decreased the expression of VEGFA and two other angiogenesis markers ANG1 and FLK1, while downregulation of miR-199a-5p expression *via* a miR-199a-5p inhibitor dramatically increased the expression of these three targets (**Figures 4G–I**). These results support bioinformatics prediction and confirm that VEGFA was indeed a direct target of miR-199a-5p in the HUVECs.

### MiR-199a-5p Inhibits HUVEC Migration and Tube Formation

**Figure 5:**
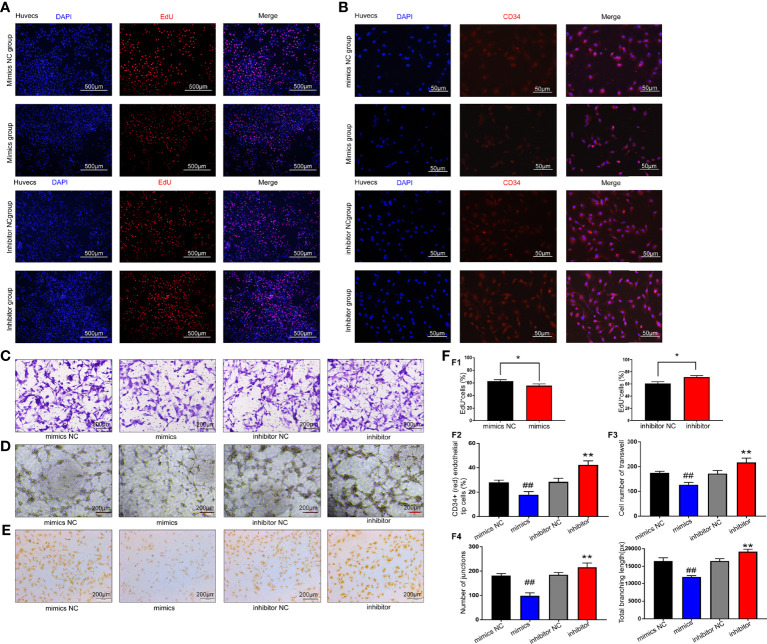
Results of miR-199a-5p functional experiments. **(A)** EdU results showed that cell proliferation was decreased after HUVEC transfection with miR-199a-5p-mimics compared with the control group. The proliferation ability of HUVECs was improved after transfection with miR-199a-5p inhibitor. **(B)** Immunofluorescence analysis of CD34+ (red) endothelial tip cells after HUVECs were transfected with miR-199a-5p-mimics or inhibitor. Nuclei were stained with DAPI (blue). **(C)** Transwell experiment results of each group of cells. **(D)** Tube formation experiment results of each group of cells. **(E)** CD31 immunohistochemistry experiment results of each group of cells. **(F)** Experimental quantitative data. F1, Quantitative data of the EdU experiment. The percentage of EdU‐positive cells for each group was quantitated using ImageJ software. F2, Quantitative CD34 immunofluorescence experiment data. F3, Quantitative data of cell Transwell experiments. F4, Quantitative data of the tube formation ability of each group of cells. * represents a statistically-significant difference compared with the control group (P < 0.05). ** represents a statistically significant difference compared with the inhibitor-NC group (P < 0.01). ^##^ represents a statistically significant difference compared with the mimic-NC group (P <0.01).

Endothelial cell migration and tube formation are crucial steps in angiogenesis. We thus attempted to investigate the effect of exosomal miR-199a-5p on the migration and tube formation of HUVECs. First, the proliferation ability of HUVECs was detected using EdU assay after transfection with miR-199a-5p mimics or inhibitor. The data showed that cell proliferation was decreased after HUVEC transfection with miR-199a-5p-mimics compared with the control group (**Figures 5A, F**1).

A wealth of evidence indicates that CD34 is expressed not only by MSCs, but also by a variety of other nonhematopoietic cell types, including vascular endothelial progenitor cells (21). Therefore, we used CD34 as a marker for the analysis of neovascularization. As shown in **Figures 5B, F**2, the percentage of CD34-positive HUVECs was remarkably higher in the miR-199a-5p inhibitor group than in the control group, indicating increased capillary density in the miR-199a-5p inhibitor group.

In addition, the data of the Transwell experiment showed that compared with the control group, the migration ability of HUVECs was significantly reduced after transfection with miR-199a-5p mimics (**Figures 5C, F**3). The *in vitro* endothelial tube formation assay revealed that miR-199a-5p mimics remarkably inhibited tube formation of HUVECs compared with the control (**Figures 5D, F**4).

Moreover, platelet endothelial cell adhesion molecule-1 (CD31) also participates in the process of angiogenesis. After HUVECs were transfected with mimics or inhibitors, CD31 immunohistochemistry was performed. The results suggested that miR-199a-5p mimics obviously reduced the expression of CD31 in HUVECs, while the inhibitor distinctly enhanced the expression of CD31 (**Figure 5E**). In summary, miR-199a-5p suppressed the proliferation, migration and neovascularization of HUVECs.

### VEGFA SiRNA Attenuates the Proliferation, Migration and Neovascularization of HUVECs Induced by a MiR-199a-5p-Inhibitor

**Figure 6:**
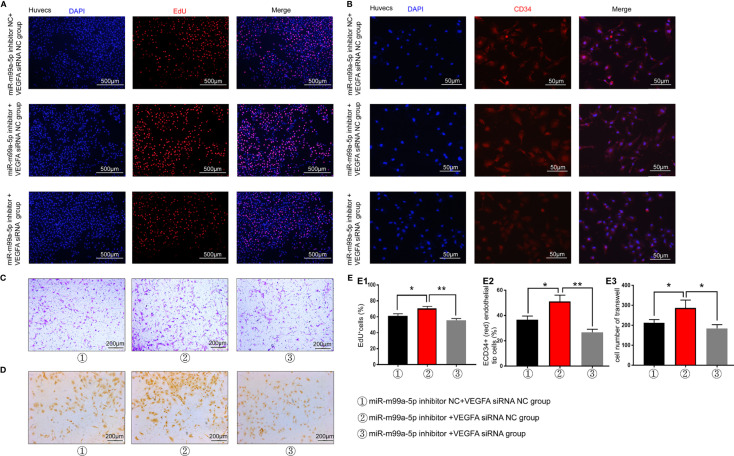
miR-199a-5p rescue experiment results. **(A)** EdU experimental results suggest that the effect of the miR-199a-5p inhibitor on cell proliferation can be reversed by VEGFA-siRNA. **(B)** The ratio of immunofluorescence analysis of CD34+ (red) endothelial tip cells was decreased after HUVECs were transfected with miR-199a-5p-inhibitor and VEGFA-siRNA. Nuclei were stained with DAPI (blue). **(C)** Transwell experiment results of each group of cells. **(D)** CD31 immunohistochemistry experiment results of each group of cells. **(E)** Experimental quantitative data. E1, Quantitative data of EdU experiment. E2, Quantitative CD34 immunofluorescence experiment data. E3, Quantitative data of cell Transwell experiments. ** represents a statistically significant difference compared with the other groups (P < 0.01). *P < 0.05.

As VEGFA was proven to be the target gene of miR-199a-5p, VEGFA siRNA was utilized for the miR-199a-5p rescue experiment. The EdU assay revealed that the promotion of HUVEC proliferation by the miR-199a-5p inhibitor could be attenuated by VEGFA-siRNA (**Figures 6A, E**). In addition, the expression of CD34 and CD31 was significantly decreased in miR-199a-5p inhibitor and VEGFA siRNA transfected HUVECs compared with miR-199a-5p inhibitor transfected HUVECs (**Figures 6B, D, E**). These data suggested that the promotion of neovascularization by the miR-199a-5p inhibitor in HUVECs could be weakened by VEGFA-siRNA. Moreover, Transwell assay results indicated that VEGFA siRNA obviously reduced the migration of HUVECs transfected with miR-199a-5p-inhibitor (**Figures 6C, E**). Above all, suppression of the expression of VEGFA weakened the promotion of HUVEC proliferation, migration and neovascularization of induced by miR-199a-5p inhibitor, indicating that the proliferation and migration of endothelial cells promoted by miR-199a-5p was mediated by VEGFA.

### MiR-199a-5p Inhibits Osteosarcoma Tumorigenesis and Angiogenesis *In Vivo*

**Figure 7:**
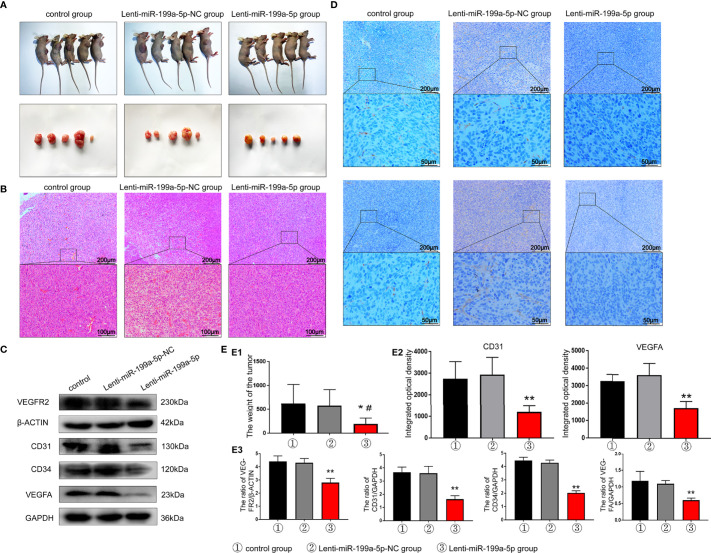
Results of tumor formation experiment in nude mice. **(A)** Photographs of nude mice and tumor in each group. **(B)** H&E staining results of tumor sections indicated that the number of tumor vascular tissues in the Lenti-miR-199a-5p group decreased. **(C)** western blot analysis showed that the protein expression levels of VEGFR2, CD31, CD34 and VEGFA protein were markedly increased in the tumor tissue of Lenti-miR-199a-5p group. **(D)** The immunohistochemical analysis of miR-199a-5p inhibited osteogenesis *in vivo*. Immunohistochemical analysis of CD31 and VEGFA was used to detect osteogenesis in the tumor sections. The brown color represents positive staining of CD31 and VEGFA. **(E)** Experimental quantitative data. E1, Comparison of tumor weight between the three groups. E2, The integrated optical density of positive staining was measured by Image-Pro Plus 6.0 software. E3, Quantitative data analysis of western blot results. ** represents P < 0.01vs other groups. *P < 0.05 represents P < 0.05 vs other groups..

To further investigate the intrinsic role of miR-199a-5p in osteosarcoma tumorigenesis and angiogenesis, *in vivo* xenograft models were established. The mouse group injected with Lenti-miR-199a-5p had a lower proliferation rate, and formed evidently smaller tumors than the Lenti-miR-199a-5p negative control (Lenti-miR-199a-5p-NC) group, as shown in **Figures 7A, B, E**1. The tumor weight at the time of death in the Lenti-miR-199a-5p group was 194.24± 122.43 g, which was significantly less than that in the Lenti-miR-199a-5p-NC group (573.06± 343.14 g). These data suggest that Lenti-miR-199a-5p reduces the tumor volume and growth rate of osteosarcoma cells *in vivo*.

To further clarify the effect of miR-199a-5p on tumor angiogenesis *in vivo*, H&E staining was used to detect the number of vascular tissues in tumor sections. The results indicated that the number of tumor vascular tissues in the Lenti-miR-199a-5p group decreased (**Figure 7B**). In addition, western blotting assays and IHC were performed to determine whether VEGFA expression was suppressed by miR-199a-5p *in vivo*, we found that the expression of VEGFA was largely inhibited by Lenti-miR-199a-5p, and later, VEGFR2, CD31, and CD34 were also detected (**Figures 7C**). VEGFA and CD31 staining revealed that HUVECs were significantly decreased in mice of the Lenti-miR-199a-5p group compared with those of the Lenti-miR-199a-5p-NC group, indicating that miR-199a-5p attenuated angiogenesis (**Figures 7D, E**2-3). Taken together, these results indicate that the overexpression of miR-199a-5p *via* lentiRNA treatment leads to an inhibitory growth and tumor angiogenesis in osteosarcoma.

## Discussion

Numerous reports have revealed that miRNAs can function as tumor promotors or tumor suppressors and regulate the tumorigenesis and development of cancers (22). miR‐199a‐5p has been studied in a variety of cancers, and has been shown to modulate the tumor microenvironment *via* exosomes (23). However, until now, the osteosarcoma cell-secreted exosomal miR-199a-5p interaction with VEGFA in osteosarcoma has not been explored, especially in relation to angiogenesis. The current study revealed that through systematic screening, we identified that miR-199a-5p was markedly downregulated in osteosarcoma plasma samples. In addition, HUVEC proliferation, migration, invasion and new vessel formation were significantly inhibited by miR-199a-5p both *in vitro* and *in vivo*. Furthermore, miR-199a-5p suppressed the transduction of the VEGFA signaling pathway. Therefore, miR-199a-5p could be identified as a tumor-suppressing factor in osteosarcoma proliferation and metastasis.

miRNAs can transfer between tumor cells and stromal cells such as vascular endothelial cells, thereby effectively silencing mRNA to reprogram the target cell transcriptome (24, 25). For instance, cancer-derived exosomal miR-25-3p promotes premetastatic niche formation by inducing vascular permeability and angiogenesis in colorectal cancer (25). In addition, miR-205 derived from ovarian cancer is secreted into the extracellular space and efficiently transferred to adjacent endothelial cells in an exosome-dependent manner, thereby inducing angiogenesis and promoting tumor metastasis (26).

Previous studies have indicated that miR-199a-5p is considered a tumor promotor in hepatocellular carcinoma (12) and a tumor suppressor in ovarian cancer (13) and glioma (14). However, the role of miR-199a-5p in osteosarcoma is still controversial. Chen Wang et al. reported that miR-199a-5p promotes tumor growth by dual-targeting PIAS3 and p27 in human osteosarcoma (27). On the other hand, it was suggested that miR-199a-5p was at a low level in osteosarcoma cells, and miR-199a-5p overexpression was associated with decreased proliferation and increased apoptosis of osteosarcoma cells (28). This contradiction may be related to the limited number of clinical samples selected in the two studies and the pathological types of tissue samples. In our study, we discovered that miR-199a-5p was notably downregulated in osteosarcoma cells and osteosarcoma plasma samples compared with normal plasma samples. *in vitroin vivo*Additionally, miR-199a-5p can be transported from osteosarcoma cells to HUVECs through exosomes, thereby targeting VEGFA and inhibiting cell proliferation, migration and angiogenesis *in vitro* and *in vivo*. These results indicated that miR-199a-5p, which contains tissue heterogeneity, is an intrinsic antioncogene in human osteosarcoma.

In addition, the expression of miR-199a-5p and VEGFA in osteosarcoma cells was negatively correlated. VEGFA is a well-known regulator of human tumor angiogenesis and metastasis. Metastasis is the main reason for the poor prognosis of patients with osteosarcoma. Metastasis and blood vessels usually coexist with each other and share many characteristics, including molecular pathways (29, 30). In the present study, a HUVEC assay was used to further determine whether miR-199a-5p could affect angiogenesis by targeting VEGFA. We found that miR-199a-5p inhibited the cell migration and tube-forming ability of HUVECs *in vitro*.

In conclusion, in this work, the potential clinical relevance of miR-199a-5p in osteosarcoma was identified. In addition, miR-199a-5p can be transported from osteosarcoma cells to HUVECs through exosomes, thereby targeting VEGFA and inhibiting the growth and angiogenesis of osteosarcoma. Taken together, miR-199a-5p may act as a biomarker in the diagnosis of osteosarcoma.

## Data Availability Statement

The original contributions presented in the study are included in the article/**Supplementary Material**. Further inquiries can be directed to the corresponding author.

## Ethics Statement

The animal study was reviewed and approved by Experimental Animal Welfare and Ethics Review Committee of Shandong University Qilu Hospital.

## Author Contributions

LZ contributed to the conceptualization, methodology and original draft writing. HC contributed to the data analysis and review, editing and writing of the manuscript. GG contributed software. DH was responsible for the validation. YY and XL contributed to the formal analysis. YC was responsible for the supervision. GJ was responsible for the project administration and funding acquisition. All authors contributed to the article and approved the submitted version.

## Funding

This study was supported by the National Natural Science Foundation of China (NSFC) (Grant numbers 81602361, 81702261), China postdoctoral science foundation (Grant number 2018M642668), and Natural Science Foundation of Shandong Province (ZR2021MH293).

## Conflict of Interest

The authors declare that the research was conducted in the absence of any commercial or financial relationships that could be construed as a potential conflict of interest.

## Publisher’s Note

All claims expressed in this article are solely those of the authors and do not necessarily represent those of their affiliated organizations, or those of the publisher, the editors and the reviewers. Any product that may be evaluated in this article, or claim that may be made by its manufacturer, is not guaranteed or endorsed by the publisher.

## Supplementary Material

Supplementary Figure S1 The overall survival analysis of the predicted targets of miR-663a, miR-199a-5p and miR-223-3p from the R2 database (https://hgserver1.amc.nl/cgi-bin/r2/main.cgi).

Supplementary Figure S2 The metastasis free survival analysis of the predicted targets of miR-663a, miR-199a-5p and miR-223-3p from the R2 database (https://hgserver1.amc.nl/cgi-bin/r2/main.cgi).

Supplementary Figure S3 Electron microscope pictures of exosomes.

Supplementary Figure S4 Relative expression of miR-199a-5p in HUVECs after co-culture with HOS-exosome.
