# Supplementary figures and images for: Exosomal MiR-199a-5p Inhibits Tumorigenesis and Angiogenesis by Targeting VEGFA in Osteosarcoma
Source: Front Oncol. 2022 May 16;12:884559. doi: 10.3389/fonc.2022.884559 (PMC9148962; doi:10.3389/fonc.2022.884559)

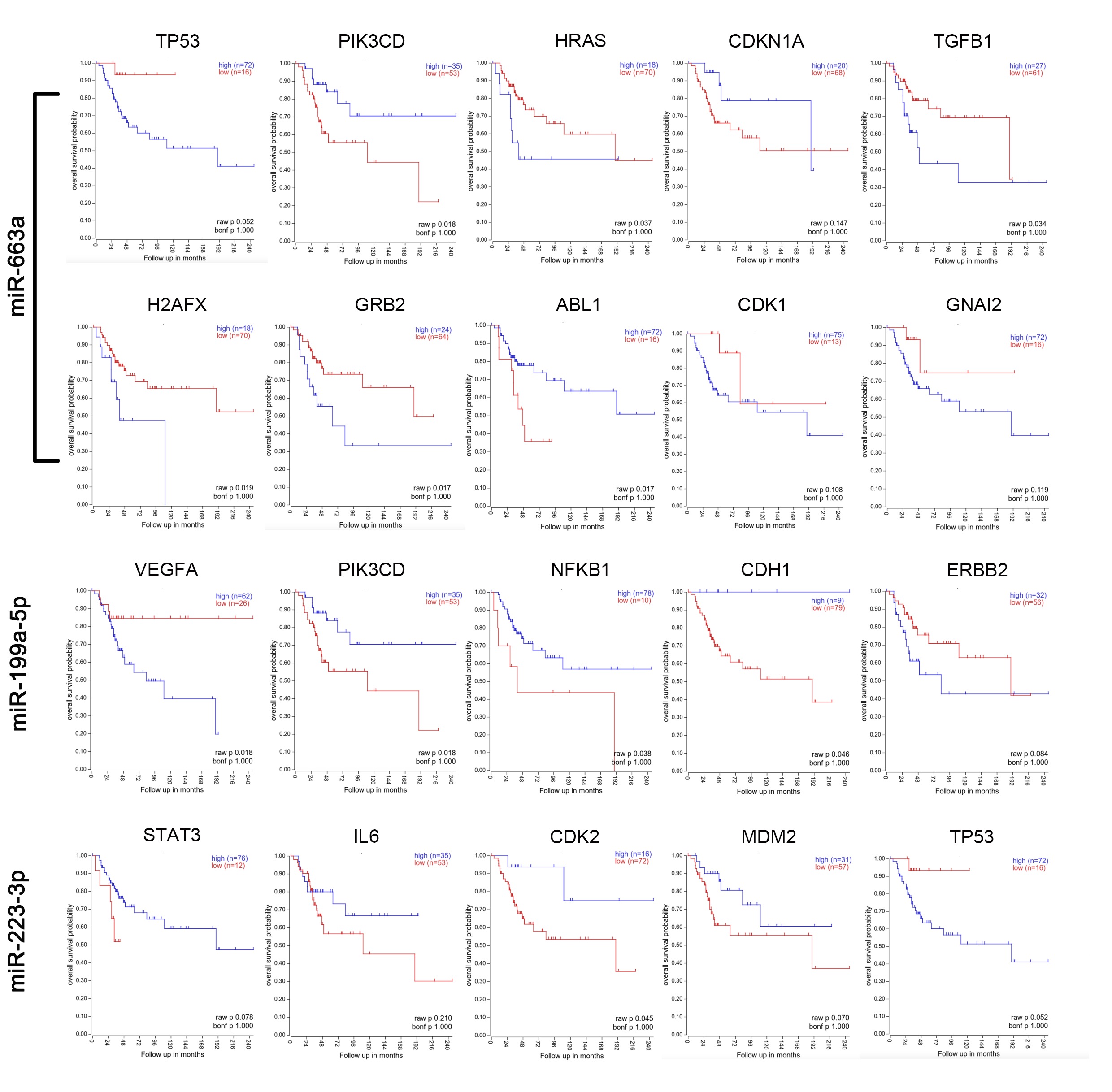

Supplement: Supplementary Figure S1 — The overall survival analysis of the predicted targets of miR-663a, miR-199a-5p and miR-223-3p from the R2 database (https://hgserver1.amc.nl/cgi-bin/r2/main.cgi). [file Image_1.jpg]

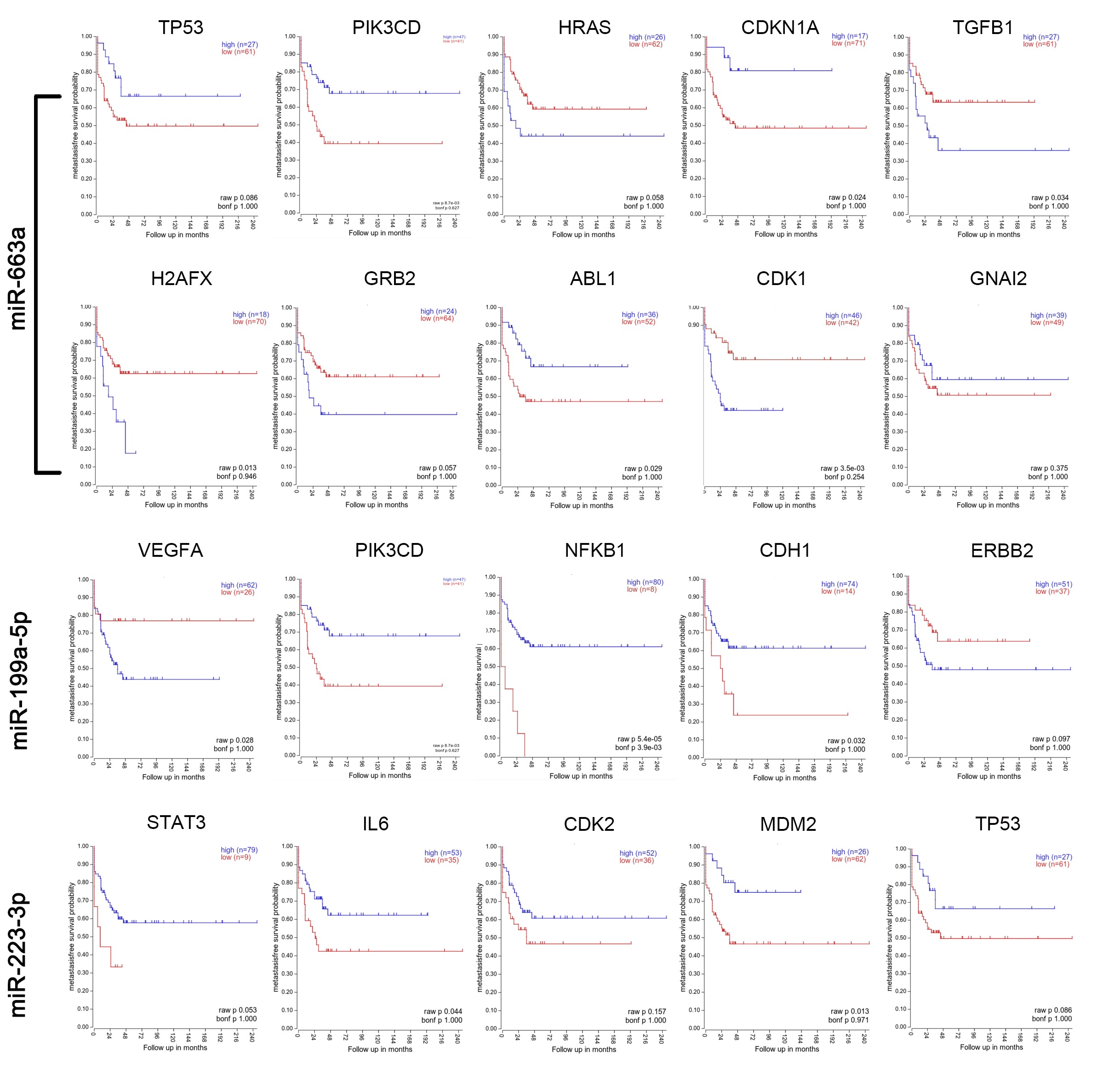

Supplement: Supplementary Figure S2 — The metastasis free survival analysis of the predicted targets of miR-663a, miR-199a-5p and miR-223-3p from the R2 database (https://hgserver1.amc.nl/cgi-bin/r2/main.cgi). [file Image_2.jpg]

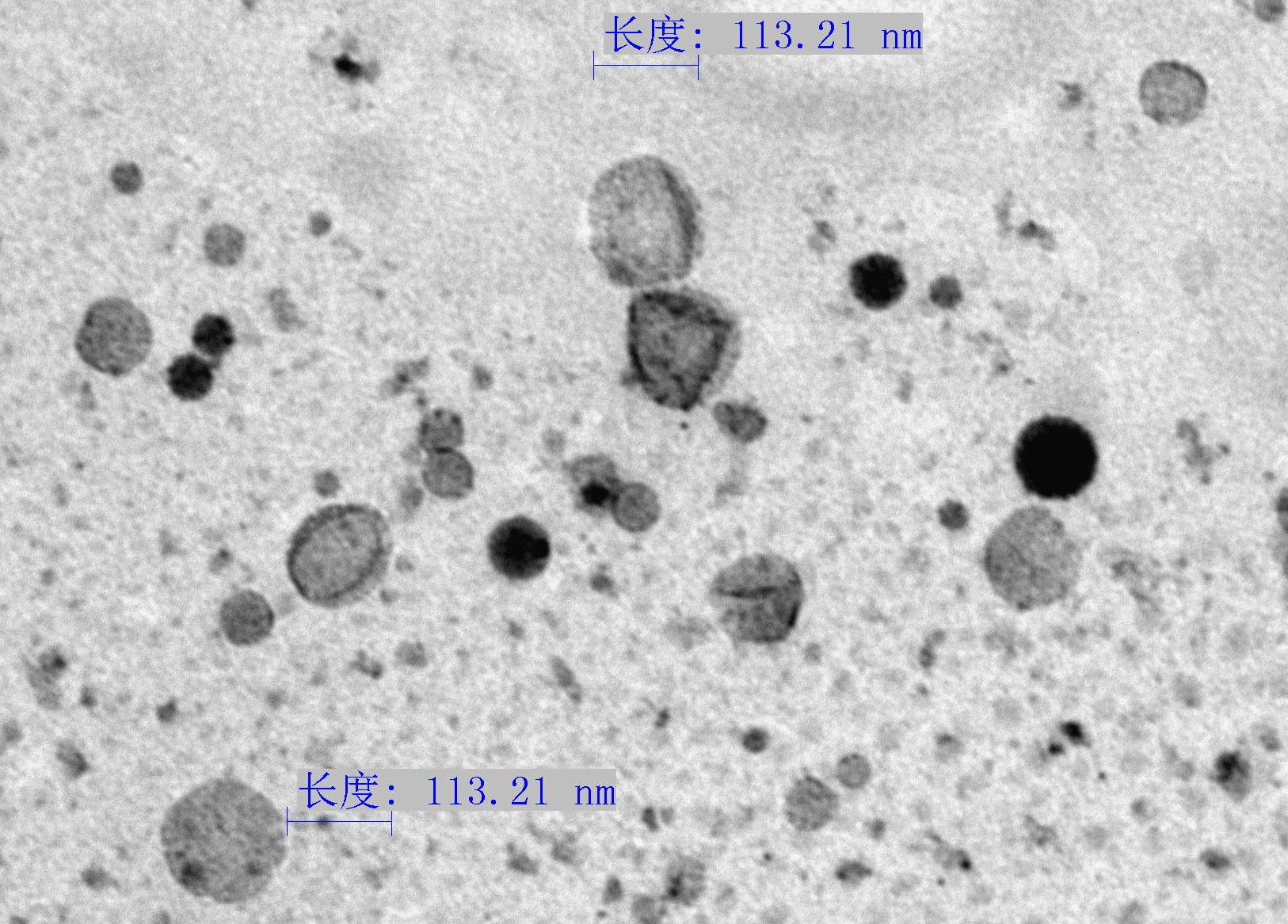

Supplement: Supplementary Figure S3 — Electron microscope pictures of exosomes. [file Image_3.jpg]

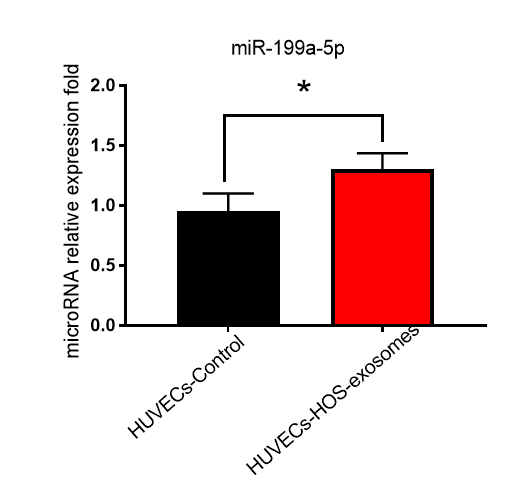

Supplement: Supplementary Figure S4 — Relative expression of miR-199a-5p in HUVECs after co-culture with HOS-exosome. [file Image_4.tif]
